# Supplementary material for: Comparative analysis of bacterial communities associated with healthy and diseased corals in the Indonesian sea
Source: PeerJ. 2019 Dec 19;7:e8137. doi: 10.7717/peerj.8137 (PMC6925950; doi:10.7717/peerj.8137)
Supplement: Supplemental Information 1 [file peerj-07-8137-s001.docx]

**Table S1:** ANOVA analysis on number of sequences obtained from individual coral species

| **ANOVA:number of sequence** | | **95% Confidence Interval for Mean** | | | | | | | |
| --- | --- | --- | --- | --- | --- | --- | --- | --- | --- |
|  |  | **N** | **Mean** | **Std. Deviation** | **Std. Error** | **Lower Bound** | **Upper Bound** | **Minimum** | **Maximum** |
| No. sequence | AcAs | 3 | 66136 | 39387.74 | 22740.52 | -31708.57 | 163980.57 | 24556 | 102886 |
|  | AcAsInf | 3 | 65685.67 | 40164.06 | 23188.73 | -34087.4 | 165458.74 | 27701 | 107722 |
|  | AcFo | 3 | 70133 | 36618.76 | 21141.85 | -20833.05 | 161099.05 | 27979 | 94076 |
|  | AcFoInf | 3 | 53274 | 27843.5 | 16075.45 | -15893.09 | 122441.09 | 21453 | 73163 |
|  | Cy | 3 | 55305 | 21850.53 | 12615.41 | 1025.28 | 109584.72 | 33475 | 77176 |
|  | CyInf | 3 | 47865 | 21147.41 | 12209.46 | -4668.08 | 100398.08 | 23850 | 63703 |
|  | Is | 3 | 87233.33 | 27514.82 | 15885.69 | 18882.73 | 155583.94 | 55471 | 103771 |
|  | IsInf | 3 | 86410.33 | 7005.03 | 4044.36 | 69008.87 | 103811.8 | 78463 | 91688 |
|  | Total | 24 | 66505.29 | 28350.66 | 5787.06 | 54533.86 | 78476.73 | 21453 | 107722 |
|  |  |  |  |  |  |  |  |  |  |
| **Table: ANOVA** |  |  |  |  |  |  |  |  |  |
|  |  | **Sum of Squares** | **df** | **Mean Square** | **F** | **Sig.** |  |  |  |
| No. sequence | Between Groups | 4463414731 | 7 | 637630675.9 | 0.73 | **0.652** |  |  |  |
|  | Within Groups | 14023069222 | 16 | 876441826.4 |  |  |  |  |  |
|  | Total | 18486483953 | 23 |  |  |  |  |  |  |
