# Supplementary figures and images for: Comparative analysis of bacterial communities associated with healthy and diseased corals in the Indonesian sea
Source: PeerJ. 2019 Dec 19;7:e8137. doi: 10.7717/peerj.8137 (PMC6925950; doi:10.7717/peerj.8137)

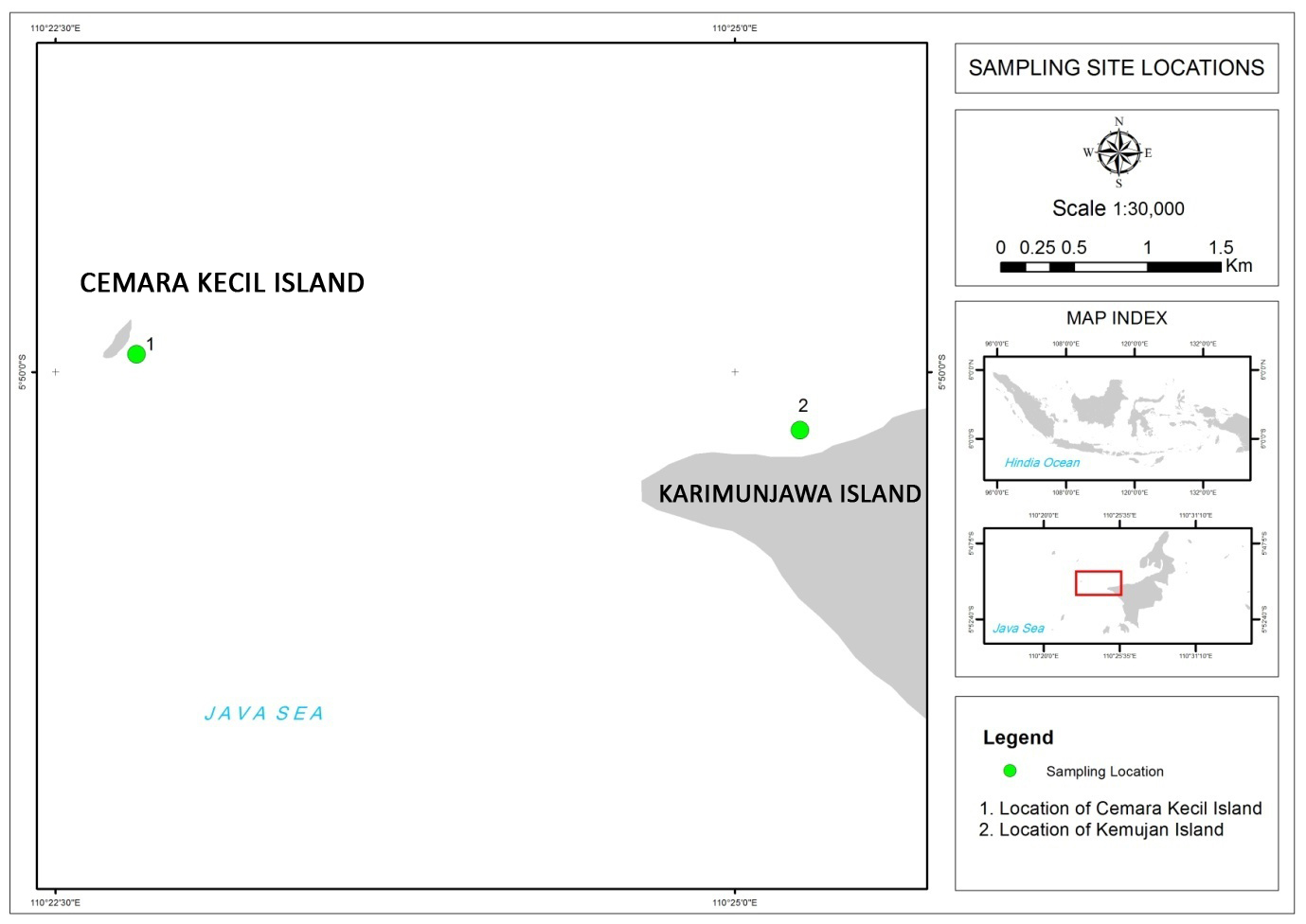

Supplement: Figure S1 [file peerj-07-8137-s002.png]

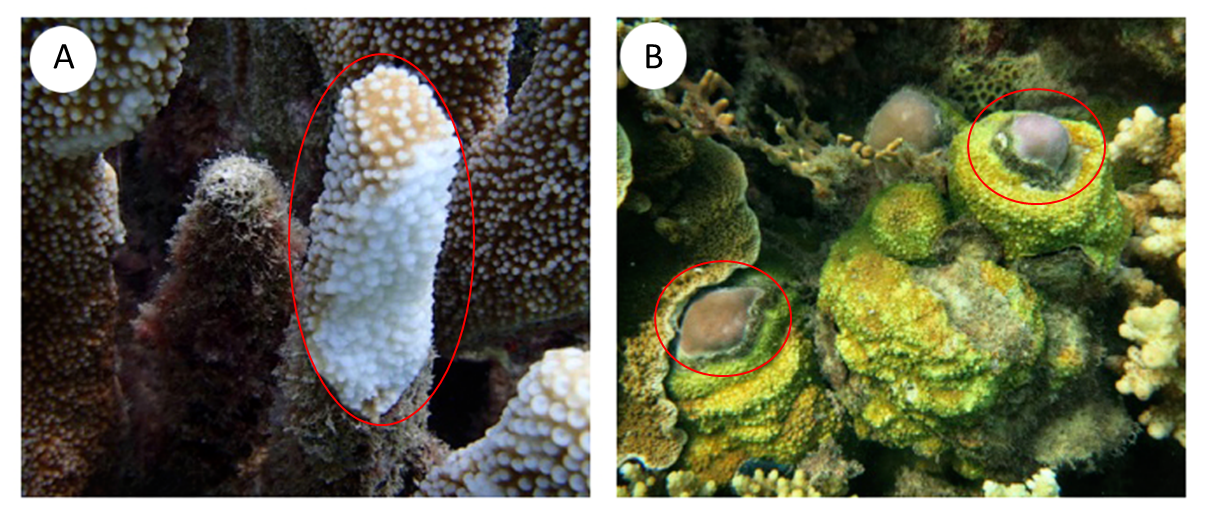

Supplement: Figure S2 — These two corals have found with limited distribution in Cemara Kecil Islands where the samples were collected (S 05° 49′57,7″E 110°22′50,5″). The Isopora sp. has column shape colony with small rounded corallites form cluster on the surface without axial corallites on the branch tips. Cyphastrea sp. has corallites low cones with separated wall and contains beaded spinules cover surface between corallites. The physical appearances of the healthy and diseased parts of these two coral species are shown for comparison with the diseased parts circled. Isopora sp. infected by white plaque disease showed progressing band of bleached coral tissue followed by necrotic tissue starting from the base of the branch. Cyphastrea sp. infected by yellow blotch disease resulted in a band of yellow tissue around the enlarging sediment patch. [file peerj-07-8137-s003.png]

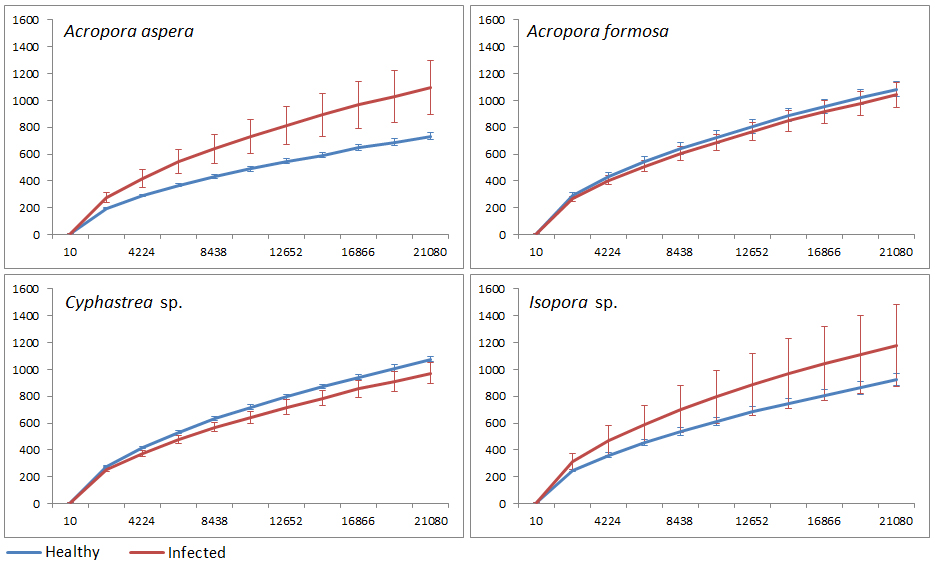

Supplement: Figure S3 — The dataset were normalized for the number of sequences obtained from individual coral species with different health status. The numbers of unique OTUs were clustered at 3% sequence dissimilarities. [file peerj-07-8137-s004.png]

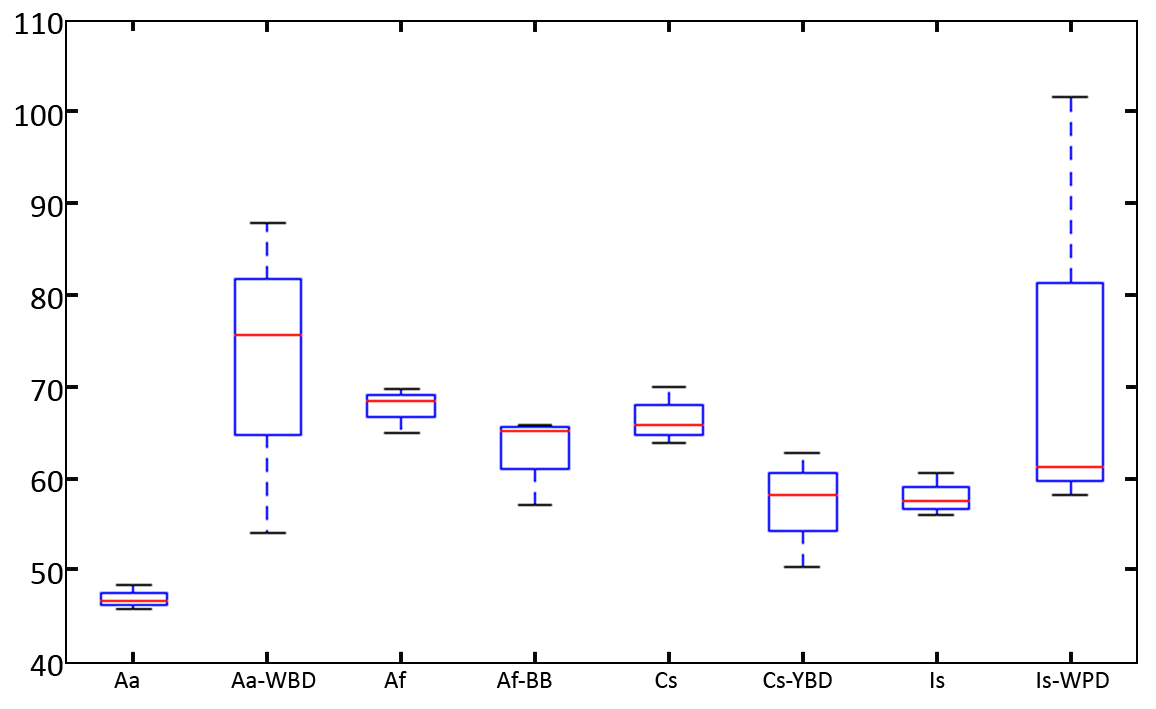

Supplement: Figure S4 [file peerj-07-8137-s005.png]
